# A Splice Variant of the *MYH7* Gene Is Causative in a Family with Isolated Left Ventricular Noncompaction Cardiomyopathy

**DOI:** 10.3390/genes13101750

**Published:** 2022-09-28

**Authors:** Roman P. Myasnikov, Olga V. Kulikova, Alexey N. Meshkov, Anna A. Bukaeva, Anna V. Kiseleva, Alexandra I. Ershova, Anna V. Petukhova, Mikhail G. Divashuk, Evgenia D. Zotova, Evgeniia A. Sotnikova, Alexandra A. Abisheva, Alisa V. Muraveva, Sergey N. Koretskiy, Sergey V. Popov, Marina V. Utkina, Ekaterina A. Snigir, Sergey I. Mitrofanov, Ksenia D. Konureeva, Elena A. Mershina, Valentin E. Sinitsyn, Sergey M. Yudin, Oxana M. Drapkina

**Affiliations:** 1 National Medical Research Center for Therapy and Preventive Medicine, 101990 Moscow, Russia; 2 FSBI “National Medical Research Center of Endocrinology” of the Ministry of Health of Russia, 115478 Moscow, Russia; 3 All-Russia Research Institute of Agricultural Biotechnology, Timiryazevskaya Street, 42, 127550 Moscow, Russia; 4 Federal State Budgetary Institution “Centre for Strategic Planning and Management of Biomedical Health Risks” of the Federal Medical Biological Agency, 119121 Moscow, Russia; 5 Medical Research and Educational Center, Lomonosov Moscow State University, 119991 Moscow, Russia

**Keywords:** left ventricular noncompaction, genetic testing, *MYH7*, splicing

## Abstract

Variants of the *MYH7* gene have been associated with a number of primary cardiac conditions, including left ventricular noncompaction cardiomyopathy (LVNC). Most cases of *MYH7*-related diseases are associated with such variant types as missense substitutions and in-frame indels. Thus, truncating variants in *MYH7* (*MYH7*tv) and associated mechanism of haploinsufficiency are usually considered not pathogenic in these disorders. However, recent large-scale studies demonstrated evidence of the significance of *MYH7*tv for LVNC and gave rise to an assumption that haploinsufficiency may be the causal mechanism for LVNC. In this article, we present a family with isolated LVNC and a heterozygous splice variant of the *MYH7* gene, analyze possible consequences of this variant and conclude that not all variants that are predicted truncating really act through haploinsufficiency. This study can highlight the importance of a precise assessment of *MYH7* splicing variants and their participation in the development of LVNC.

## 1. Introduction

Left ventricular noncompaction cardiomyopathy (LVNC) is characterized by the presence of a “spongy” hypertrabeculated endocardial layer of the heart wall. During recent years, along with the improvement of instrumental diagnostics, the detectability of LVNC has been growing. First by discovering the high variability of its clinical presentation, and second by highlighting the need for genetic testing in order to avoid excessive diagnostics. It is now recognized that genetic diagnostics is necessary for correct clinical management and risk stratification in patients with LVNC [1]. At the same time, extreme genetic and phenotypic overlapping of LVNC and other primary cardiomyopathies makes this task challenging. 

Many genes have been reported in connection with LVNC [2], but only few of them show robust association, confirmed by multiple studies. Almost all such genes encode sarcomere proteins and are associated with other types of primary cardiomyopathies [1]. One such gene, *MYH7* (OMIM #160760), is responsible for hypertrophic cardiomyopathy (HCM), dilated cardiomyopathy (DCM), left ventricular noncompaction (LVNC), Laing distal myopathy, and myosin storage myopathy [3,4,5]. 

During recent years, growing efforts are being made to distinguish the LVNC-causing genetic variants from those responsible for other types of cardiomyopathies. Recent results in this area of genetic research draw attention to predicted truncating variants in the *MYH7* gene (*MYH7*tv), which include nonsense substitutions, frameshifts, and splice donor/acceptor variants [6].

Some splice variants do not lead to a premature stop codon and, therefore, do not result in haploinsufficiency. These splice variants cause exon skipping without frame shift [7] and lead to a dominant negative effect identical to that of missenses and in-frame indels, the prevalent types of disease-causing alterations in *MYH7* [8,9]. In-frame exon skipping was previously described in *MYH7*-related myopathic conditions [10], but not in primary cardiomyopathies. However, more recent studies demonstrate that the role of *MYH7*tv in LVNC pathogenesis may be underestimated. 

In this article, we present a family of two generations with isolated LVNC and a heterozygous variant altering a canonical splice site in the *MYH7* gene. Bioinformatics analysis showed that the most likely outcome of the canonical donor splice site variant, skipping of exon 10, would result in translation of an aberrant protein product missing 33 amino acid residues, rather than premature truncation. This study presents an example of this pathogenetic mechanism in LVNC and will highlight the importance of correct estimation of genetic variants that are predicted truncation.

## 2. Materials and Methods

### 2.1. Clinical Investigation of the Patients

Two generations of a family with LVNC were admitted at the National Medical Research Center for Therapy and Preventive Medicine (Moscow, Russia). Family members underwent clinical examination, which included blood sample collection, biochemical and general examination, electrocardiography using 24-h Holter monitoring electrocardiogram (HM-ECG), cardiac magnetic resonance imaging (cMRI), and echocardiography (ECHO) with contrast. ECHO and cMRI imaging criteria of LVNC were applied, as previously suggested by Jenni et al. [11] and Petersen et al. [12]. Our study was conducted in accordance with the Declaration of Helsinki in its current form and approved by the Institutional Review Boards of the National Medical Research Center for Therapy and Preventive Medicine (Moscow, Russia). Every participant and/or their legal representative gave their written informed consent to be involved in this study.

### 2.2. Cardiac Magnetic Resonance Imaging

cMRI was performed with a 1.5-T imager (Magnetom Avanto, Siemens, Munich, Germany) using a standard protocol. Breath-hold cine MRI was performed using ECG-gated segmented true fast imaging with steady-state free-precession (SSFP). Cine MRI was acquired in long-axis and short-axis planes covering the whole LV and right ventricle (RV). Late gadolinium enhancement (LGE) images were acquired in the same planes 15 min after IV injection of the gadolinium contrast agent (Gd–DTPA–BMA, Omniscan, GE Healthcare Inc., Chicago, IL, USA) in a dose of 0.15 mmol/kg using inversion-recovery turbo fast low-angle shot (FLASH) pulse sequence.

### 2.3. Whole Genome Sequencing and Bioinformatic Analysis

Molecular genetic analysis was performed in the Centre for Strategic Planning and Management of Biomedical Health Risks (Moscow, Russia) as detailed by Meshkov et al. [13]. We extracted DNA from whole blood, then performed whole genome sequencing (WGS) on Novaseq 6000 (Illumina, San Diego, CA, USA) up to 30× coverage. After that, SNPs and long structural variants were called via Dragen Bio-IT platform (Illumina, San Diego, CA, USA) and Smoove software, respectively. Clinical interpretation of potentially clinically relevant findings was performed in accordance with current guidelines, considering the modified frameworks provided by ClinGen [14,15]. Validation of NGS results was carried out at the National Medical Research Center for Therapy and Preventive Medicine (Moscow, Russia) by Sanger sequencing using the Applied Biosystem 3500 Genetic Analyzer (Thermo Fisher Scientific, Waltham, MA, USA) in accordance with the manufacturer’s protocol. For verification by Sanger sequencing the following oligonucleotides were used: 5′ AACCAATGGCCAGCGTCTTA-3′ and 5′-TCCTTGTGCCCAAACCCTAA-3′.

## 3. Results

**Figure 1:**
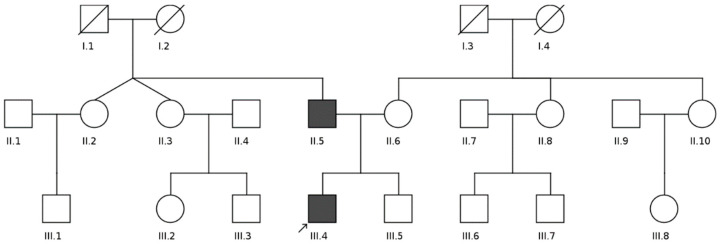
Pedigree of the family (created using the CeGaT Pedigree Chart Designer software v3.0, (CeGaT GmbH, Tübingen, Germany) [16]). Women are shown by circles and men by squares. Affected persons are shown by black figures, crossed figures indicate deceased people. The proband (III-4) is indicated by the arrow.

The clinical features of the investigated patients are summarized in the Figure 1 and Table 1 (see below).

**Figure 2:**
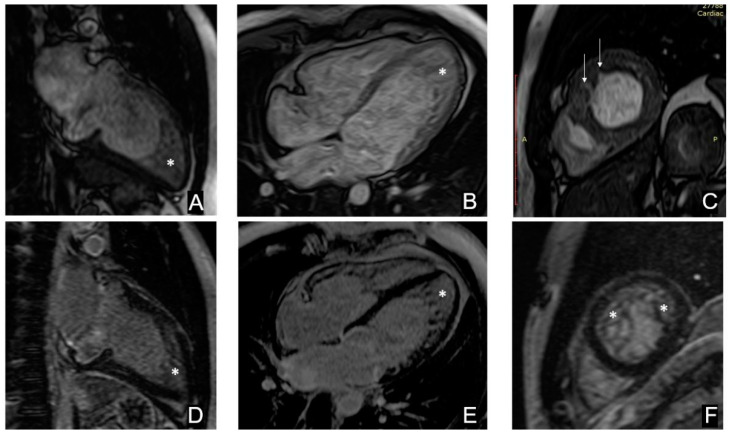
(**A**–**C**) The proband’s cardiac magnetic resonance imaging (cMRI) in cine mode, SSFP sequence: (**A**) long axis 2-chamber images, (**B**) long axis 4-chamber images, (**C**) short axis images at the level of the middle segments. (**D**–**F**) DE (delayed enhancement) cMRI images, inversion recovery (IR) sequence with suppression of the signal from the normal myocardium. No areas of contrast enhancement were detected, which indicates the absence of areas of intramyocardial fibrosis, scarring or inflammatory myocardial damage. * indicates a layer of non-compact myocardium in the apical segments, → indicates clefts in the middle anteroseptal segment.

In the proband (III-4 Table 1) mitral valve prolapse has been observed since childhood. At the age of 17, he underwent a medical examination. ECHO investigation revealed the signs of myocardial noncompaction (Jenni, Chin, and Stollberger criteria): end diastolic volume (EDV) of 157 mL, ejection fraction (EF) of 48%, and end diastolic diameter (EDD) of 5.8 cm. cMRI (Figure 2) showed two-layered structure of myocardium—the thickness of the non-compact layer was 30 mm and the thickness of the compact layer was 5 mm. The indexed left ventricular EDV was 97 mL/m^2^. In blood tests, all parameters were within normal values. B-type natriuretic peptide (BNP) level was 100 pg/mL. ECG showed sinus rhythm. According to the Holter ECG, he had a sinus rhythm with the heart rate between 45–69–130 per min without arrhythmia. The patient undergoes dynamic examination, constantly takes bisoprolol 2.5 mg/day, spironolactone 25 mg/day, and torasemide 10 mg/day.

The proband‘s father (II-5), 53 y.o., underwent a cardiological examination. According to the ECHO, the heart chambers were not expanded, with an EDD of 5.4 cm, an EF of 52%, and signs of non-compact myocardium (Jenni and Chin criteria) in the area of the apex and lateral wall. According to the HM-ECG, there were no data that suggested life-threatening cardiac arrhythmias. According to the cMRI, the heart chambers were not dilated. Signs of non-compact myocardium were present.

The proband’s 52-year-old mother (II-6) and 17-year-old brother (III-5) underwent a cardiological examination. According to the ECHO, the heart chambers were not dilated and there were no signs of non-compact myocardium.

**Figure 3:**
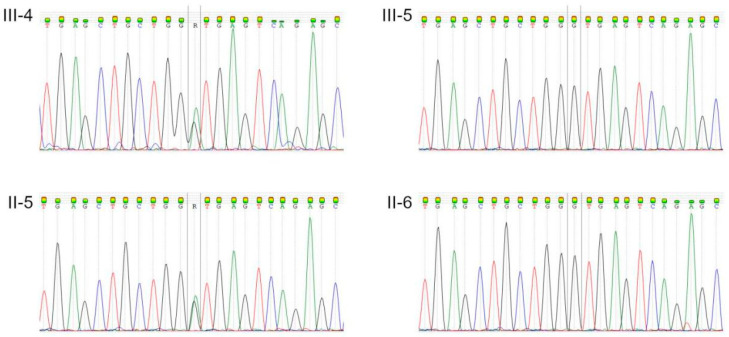
Electropherograms confirmed NM_000257.4:c.895+1G>A in the genome of patients III-4 and II-5 and a wildtype sequence in III-5 and II-6.

To establish the genetic diagnosis, WGS and bioinformatic analysis were performed for the proband and his three relatives (mother, father, and brother). We found the canonical splice site variant in the *MYH7* gene (t.NM_000257.4:c.895+1G>A; g.hg38.chr14:23430900C>T) in the proband’s and his father’s genome, but not in the genomes of other examined relatives (Figure 3). This variant is absent in gnomAD and other population databases but is reported in dbSNP [17] and in ClinVar [18]. The ClinVar entry reports it as the variant of uncertain significance (VUS), but no phenotype data are provided by the submitter. We performed the in silico splicing prediction analysis via MaxEntScan v1.0 (Christopher Burge Laboratory, Department of Biology, Massachusetts Institute of Technology, Cambridge, MA, USA) [19] and SpliceAI v1.3.1 (Kishore Jaganathan, Illumina, Inc., San Diego, CA, USA) [20] software and discovered a strong probability of donor splice site loss (see Table 2). A functional RNA study that could establish the impact of the variant on the gene and its product was not conducted due to family refusal.

## 4. Discussion

Currently, the prognosis of the disease in patients with LVNC varies from favorable to fulminant course requiring heart transplantation [21]. Here we present a family with favorable clinical course harboring the splicing variant in the *MYH7* gene.

To date, haploinsufficiency of *MYH7* is generally considered to be tolerated. ClinGen Expert Panel recommendations discourage the use of PVS1 (“very strong”) criterion of pathogenicity for predicted truncating variants in *MYH7* because such variants are thought to have only moderate impact. Thus, heterozygous predicted *MYH7*tvs, per se, are generally understood as non-causative [22].

**Figure 4:**
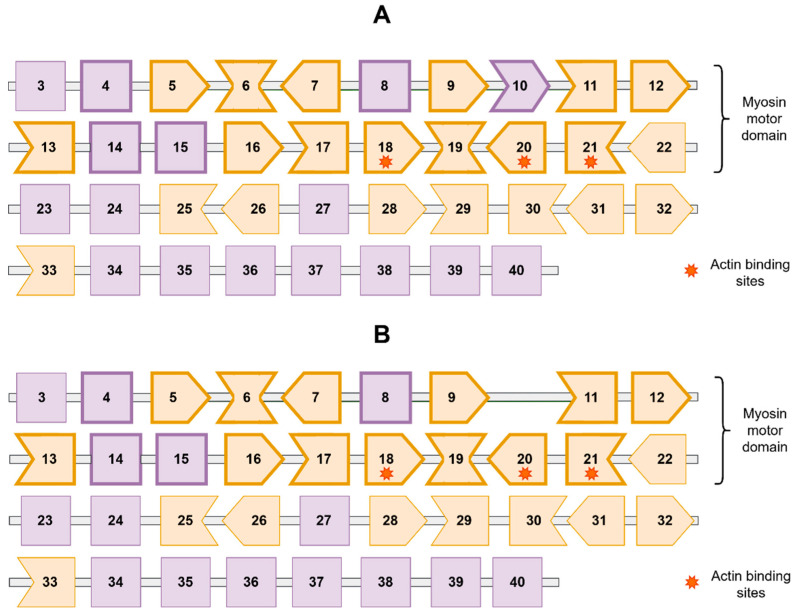
Schematic representation of exonic structure of the coding sequence of the *MYH7* gene (transcript NM_000257.4) and its predicted splicing alteration. Each polygon with a number in its center denotes the corresponding exon (exons 1 and 2 are non-coding in NM_000257.4). The shape of an exon border indicates the reading frame. In-frame exons are coloured grey and the rest are coloured yellow. Exon boundaries overtaking (+1 or −2) and lagging (−1 or +2) relative to the reading frame are shown as convex and concave, respectively. Polygons with bold contours represent the coding sequence of the myosin motor domain (exons 4-21). Orange stars indicate the sites of actin binding. (**A**): normal gene product. (**B**): predicted product with the variant t.NM_000257.4:c.895+1G>A; g.hg38.chr14:23430900C>T which was found in the patient’s genome.

The discovered variant c.895+1G>A leads to the disruption of the canonical donor splice site after exon 10 (out of 40, of which 1st and 2nd are non-coding). The analysis of the gene sequence by Mutalyzer software [23] showed that this disruption is unlikely to lead to the frame shift. Exon 10′s borders are in the same reading frame (its length is a multiple of three base pairs). We speculate that the most likely outcome of the canonical donor splice site variant, skipping of exon 10, would rather result in translation of an aberrant protein product missing 33 amino acid residues than in premature truncation (Figure 4) [7]. The affected codons encode myosin motor domain and are critically important for proper function of myosin fibrils [14]. Based on all of the above, we apply the modified PVS1_Strong pathogenicity criterion to our variant, as established in [15], and eventually evaluate this variant as likely pathogenic with the following criteria: PVS1_Strong, PM2 (low populational frequency), PP1 (familial cosegregation with the phenotype).

**Figure 5:**
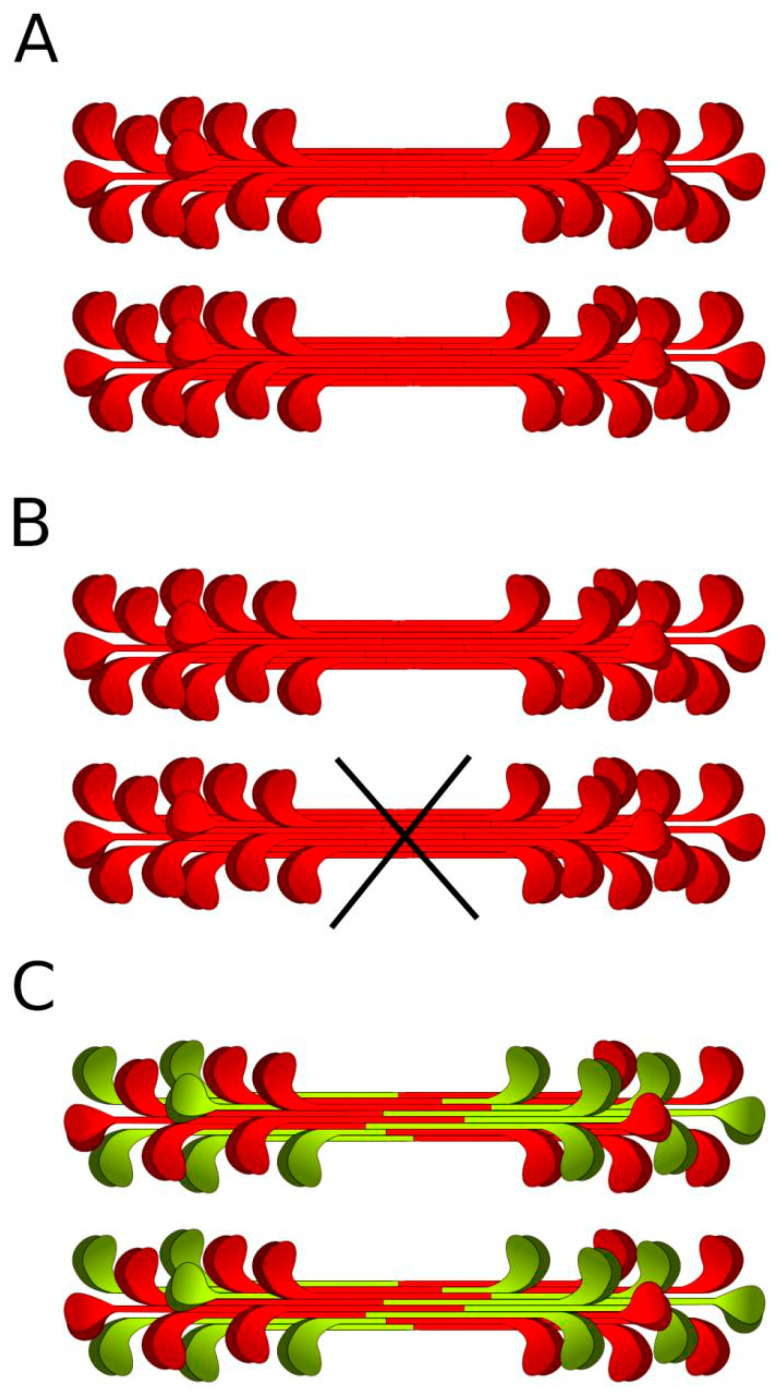
Schematic representation of how *MYH7* participates in myosin assembly. The *MYH7* gene product directly forms the myosin filament with actin-binding head. Normal functional proteins synthesized from wildtype *MYH7* are colored red. Malfunctioning proteins from mutated (by dominant negative mechanism) *MYH7* are colored green. (**A**) Normal formation of myosin filaments—synthesis of normal functional proteins from both copies of the *MYH7* gene (**B**) Haploinsufficiency—synthesis of normal functional proteins from only one copy of the gene, the total amount of filaments decreases by half, though remaining myosin is all normal. (**C**) Dominant negative mechanism—synthesis of the proteins from both copies of *MYH7*, half proteins are misfolded and malfunctioning, and all filaments contain malfunctioning proteins.

A recent large-scale study by Mazzarotto et al. [6] showed a significant prevalence of predicted truncating (i.e., nonsense, frameshift, and donor/acceptor splicing) *MYH7* variants in the cohorts of patients with LVNC. The authors compared frequencies of rare variants in six LVNC cohorts (a total of 840 patients) with population control frequencies derived from gnomAD and revealed a 20-fold enrichment of predicted *MYH7*tv in the LVNC cases versus controls. Mazzarotto et al. also found that the predicted *MYH7*tv were enriched specifically in the LVNC cases, and not enriched in the HCM or DCM cohorts. Furthermore, in ostensibly healthy controls harboring predicted *MYH7*tv, myocardial hypertrabeculation was observed. These findings suggest haploinsufficiency to be the causative mechanism for isolated noncompaction, and attract attention of clinicians to such variants. Nevertheless, it should be noted that the conclusion about the prevalence of *MYH7* haploinsufficiency in the mentioned study implied the assignment of all splicing variants to *MYH7*tv. Variation of the donor splicing region after exon 8 (c.732 splice region) was repeatedly revealed in different LVNC cohorts [6]. This region was considered a “hotspot” for *MYH7*-dependent LVNC with haploinsufficiency as a supposed pathogenetic mechanism. However, exon 8 is in-frame (Figure 4), and alterations of its donor splice site likely cause skipping of a fragment of 31 amino acids, not premature termination. This suggests that the possible mechanism of disease in c.732 splice region variants is dominant negative effect (gain of function), not haploinsufficiency (Figure 5).

The *MYH7* gene has 40 exons (38 coding), and 14 of these (Figure 4) can be skipped without disrupting the reading frame. To date, 100 donor and acceptor splice site variants in *MYH7* are present in dbSNP, with the highest minor allele frequency of 0.001647% (according to ExAC [24]), which fits the PM2 population criterion of pathogenicity of the *MYH7* variants [14]. Of them, 39 are adjacent to one of the in-frame exons and can likely lead to production of an aberrant protein product (Supplementary Table S1). However, only twelve of them are reported in ClinVar, including three pathogenic, four likely pathogenic and five VUS. Meanwhile, as evidence of possible functional roles of some variants/variant types accumulates, ClinVar entries may serve as a useful source of information for clinical interpreters, especially when there are several recordings made by different submitters. In view of emerging evidence of possible contribution of predicted *MYH7*tv (including splice variants) to LVNC, accumulation of new and existing observations in open-source databases seems essential.

The *MYH7*-associated LVNC is characterized by a relatively benign clinical course in comparison to other genetic forms of the disease. The 2018 study by Waning et al. that included 327 patients with LVNC [25] showed that the risk of decreased systolic function was higher for genotype-positive patients compared to negative ones (*p* = 0.024), but patients with variants in *MYH7* had lower probability of major adverse cardiac events compared to those with variants in other genes (*p* = 0.03). This conclusion was confirmed in the previously published work of Sedaghat-Hamedani et al., who revealed the relationship between poor prognosis in patients and variants in the *LMNA* and *RBM20* genes [26]. In patients with variants in the *TTN* and *MYH7* genes, such a correlation was not observed [26]. In our earlier study we presented a family with LVNC where the proband and his father, both harboring the novel *MYH7* missense variant, had moderate signs of systolic dysfunction and remodeling of the left ventricle, in the absence of intramyocardial fibrosis, which allowed us to classify those patients with a relatively good prognosis [27]. All these results represent the clinical features of the disease caused by *MYH7* dominant negative variants, and the phenotypes of affected family members in the present study are consistent with it. Although in absence of the functional study we cannot confidently confirm that in our case splicing variant in *MYH7* definitely leads to dominant negative effect, the concordance of clinical features with previous observations testifies to this. Co-segregation of the variant and the LVNC phenotype in two generations of the family serves as an important additional source of evidence for pathogenicity and causality of our finding.

Our clinical observations support the previously reported genotype–phenotype correlations of the *MYH7* variants and attract attention to functional diversity of splicing alterations that now appear to be underestimated. Currently, the clinical assessment of the variants in the positions corresponding to the canonical splice sites seems to be predominantly based on the widespread presumption that splicing interruption always leads to loss of one allele. Since this mechanism is not proven to date to be significant in the *MYH7*-related disorders, splicing variants tend to be interpreted as VUSes without going deep into clinical features, role of the affected exons, etc. This can be seen from the majority of the existing ClinVar entries which often contain only the postulate of non-pathogenicity of *MYH7*tv. Moreover, recent study still defines the mechanism of pathogenicity of the alterations in c.732 splice region, adjacent to the in-frame exon of *MYH7*, as “unclear” [28]. We propose that the splicing variants in *MYH7*, both known and novel, need to be systematically evaluated in terms of possible consequences of exon skipping and/or other alternative splicing events. This approach could help distinguish the truly non-causative variants from previously ignored ones.

## 5. Conclusions

Precise genetic differentiation of mechanisms underlying LVNC and its overlapping conditions is still challenging and, at the same time, important for the clinical management of affected families. Recent results suggest that truncating variants in the *MYH7* gene should not be ignored, although their role is still not entirely clear. Continuing this theme, our finding emphasizes the importance of the distinction between truncation and frame-preserving splice site variations. We believe that accurate analysis of possible molecular consequences in the patients with splicing variants in *MYH7* will expand the spectrum of known genotype-phenotype correlations and improve our knowledge of LVNC pathogenesis.

## 6. Limitations of the Study

The main limitation of the study was that the presented genetic data were based only on DNA-level experiments and in silico analysis. We were not able to validate our predictions with RNA sequencing or to conduct any functional study of alternative splicing consequences. Furthermore, we were not able to examine all relatives and could not make cMRI to the proband’s father because of his refusal.

## Figures and Tables

**Table 1:** Pedigree description.

Family Member	WGS	Sanger Sequencing	Phenotype
I-1	–	–	Died at 70
I-2	–	–	Died at 90
I-3	–	–	Died at 54, congenital heart disease, pulmonary embolism
I-4	–	–	Died at 77, enterocolitis
II-1	–	–	60 y.o, not examined
II-2	–	–	60 y.o., hypertension
II-3	–	–	60 y.o., hypertension
II-4	–	–	61 y.o., not examined
II-5	+	+	53 y.o., LVNC
II-6	+	+	52 y.o., healthy
II-7–II-10; II-8–III-3	–	–	Not examined
III-4	+	+	22 y.o., LVNC, heart failure
III-5	+	+	17 y.o., healthy
III-6–III-8	–	–	Not examined

LVNC—left ventricular noncompaction; WGS—whole genome sequencing; y.o.—years old.

**Table 2:** Values of the bioinformatic splicing predictors used for the analysis of the variant of interest (NM_000257.4:c.895+1G>A). According to MaxEntScan, the alternative allele splice site is 15.9 times weaker than the reference allele splice site (8.731 versus 0.549, value of the MaxEntScan_diff parameter is 8.182). SpliceAI predicts the loss of the donor splice site in the next position downstream the variant with a probability of 99%.

Splicing Prediction Parameter	MaxEntScan_ref	MaxEntScan_alt	MaxEntScan_diff	SpliceAI_pred_DS_DL	SpliceAI_pred_DP_DL
Value	8.731	0.549	8.182	0.99	1

## Data Availability

The data presented in this study are available on request from the corresponding author.

## Supplementary Materials

The following supporting information can be downloaded at: https://www.mdpi.com/article/10.3390/genes13101750/s1, Table S1: Currently known variants altering canonical splicing sites in the *MYH7* gene.
